# Supplementary material for: Clustered Regularly Interspaced Short Palindromic Repeats in Xanthomonas citri—Witnesses to a Global Expansion of a Bacterial Pathogen over Time
Source: Microorganisms. 2022 Aug 26;10(9):1715. doi: 10.3390/microorganisms10091715 (PMC9504256; doi:10.3390/microorganisms10091715)
Supplement: Supplementary file 1 [file microorganisms-10-01715-s001.zip › microorganisms-1839073-supplementary/Figure_S1.pptx]

## Slide 1
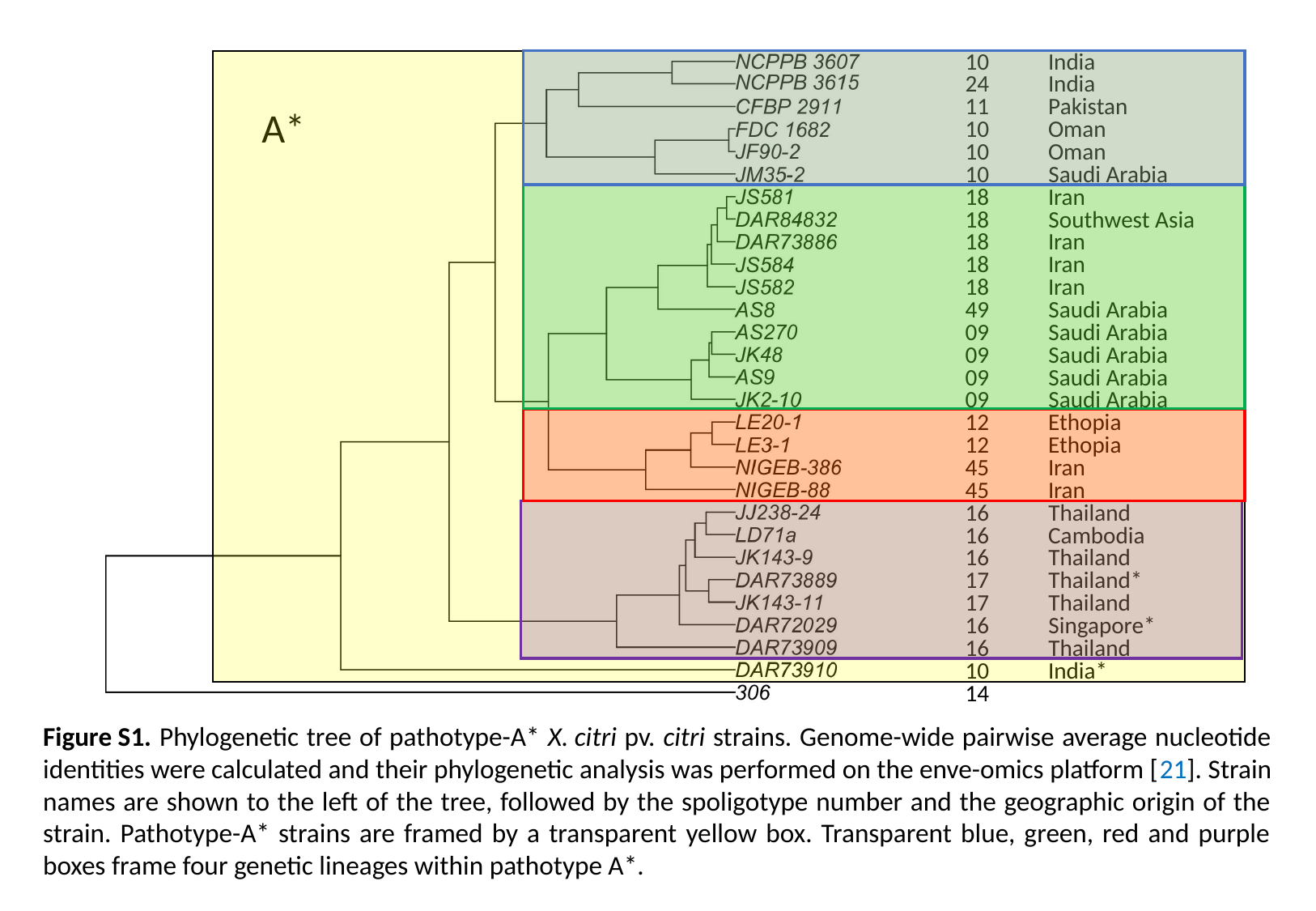

10
24
11
10
10
10
18
18
18
18
18
49
09
09
09
09
12
12
45
45
16
16
16
17
17
16
16
10
14
India
India
Pakistan
Oman
Oman
Saudi Arabia
Iran
Southwest Asia
Iran
Iran
Iran
Saudi Arabia
Saudi Arabia
Saudi Arabia
Saudi Arabia
Saudi Arabia
Ethopia
Ethopia
Iran
Iran
Thailand
Cambodia
Thailand
Thailand*
Thailand
Singapore*
Thailand
India*
A*
Figure S1. Phylogenetic tree of pathotype-A* X. citri pv. citri strains. Genome-wide pairwise average nucleotide identities were calculated and their phylogenetic analysis was performed on the enve-omics platform [21]. Strain names are shown to the left of the tree, followed by the spoligotype number and the geographic origin of the strain. Pathotype-A* strains are framed by a transparent yellow box. Transparent blue, green, red and purple boxes frame four genetic lineages within pathotype A*.
