# Supplementary material for: Clustered Regularly Interspaced Short Palindromic Repeats in Xanthomonas citri—Witnesses to a Global Expansion of a Bacterial Pathogen over Time
Source: Microorganisms. 2022 Aug 26;10(9):1715. doi: 10.3390/microorganisms10091715 (PMC9504256; doi:10.3390/microorganisms10091715)
Supplement: Supplementary file 1 [file microorganisms-10-01715-s001.zip › microorganisms-1839073-supplementary/Figure_S2.pptx]

## Slide 1
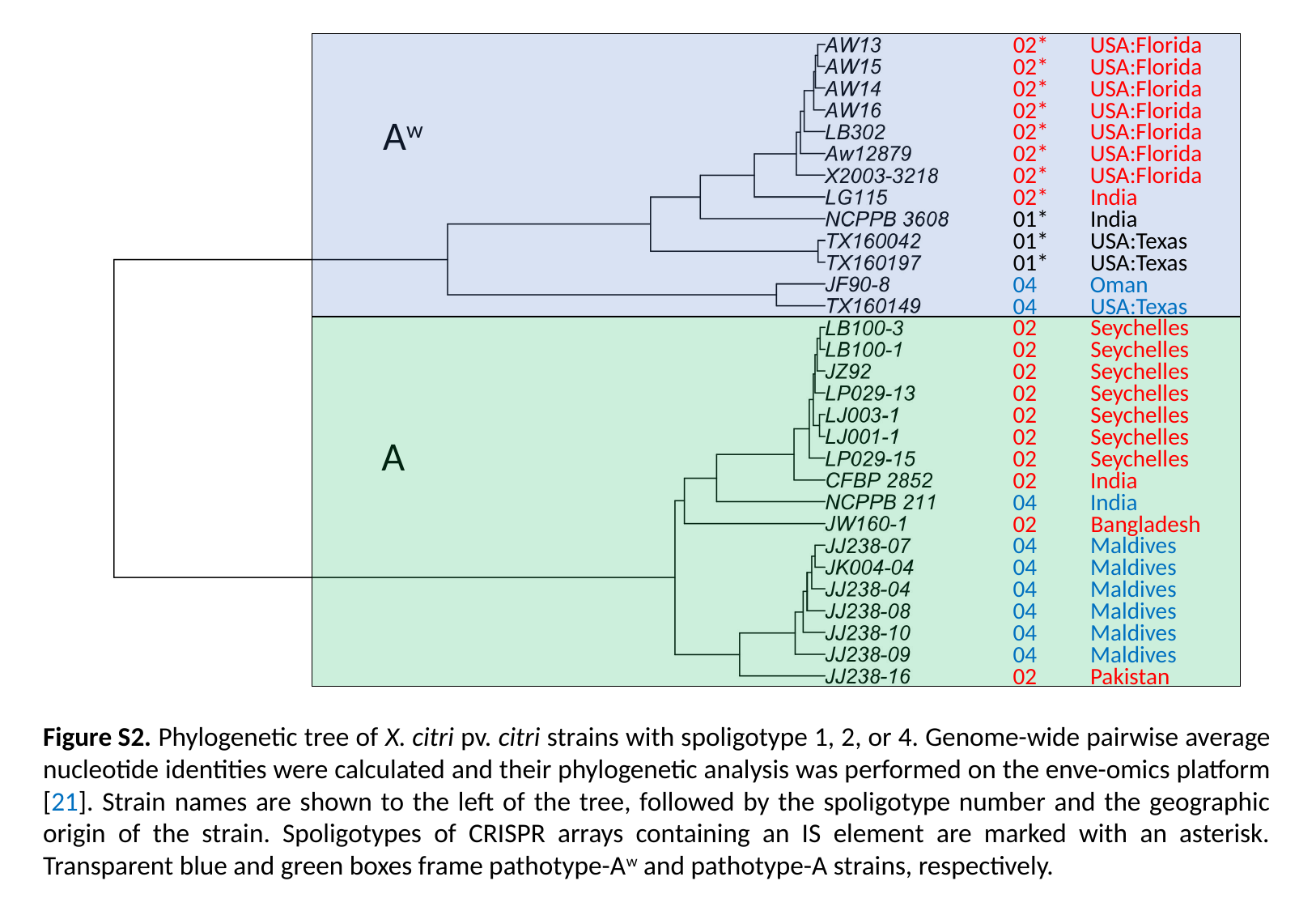

02*
USA:Florida
USA:Florida
USA:Florida
USA:Florida
USA:Florida
USA:Florida
USA:Florida
India
India
USA:Texas
USA:Texas
Oman
USA:Texas
Seychelles
Seychelles
Seychelles
Seychelles
Seychelles
Seychelles
Seychelles
India
India
Bangladesh
Maldives
Maldives
Maldives
Maldives
Maldives
Maldives
Pakistan
02*
02*
02*
Aw
02*
02*
02*
02*
01*
01*
01*
04
04
02
02
02
02
02
02
A
02
02
04
02
04
04
04
04
04
04
02
Figure S2. Phylogenetic tree of X. citri pv. citri strains with spoligotype 1, 2, or 4. Genome-wide pairwise average nucleotide identities were calculated and their phylogenetic analysis was performed on the enve-omics platform [21]. Strain names are shown to the left of the tree, followed by the spoligotype number and the geographic origin of the strain. Spoligotypes of CRISPR arrays containing an IS element are marked with an asterisk. Transparent blue and green boxes frame pathotype-Aw and pathotype-A strains, respectively.
