# Supplementary material for: Clustered Regularly Interspaced Short Palindromic Repeats in Xanthomonas citri—Witnesses to a Global Expansion of a Bacterial Pathogen over Time
Source: Microorganisms. 2022 Aug 26;10(9):1715. doi: 10.3390/microorganisms10091715 (PMC9504256; doi:10.3390/microorganisms10091715)
Supplement: Supplementary file 1 [file microorganisms-10-01715-s001.zip › microorganisms-1839073-supplementary/Figure_S3.pptx]

## Slide 1
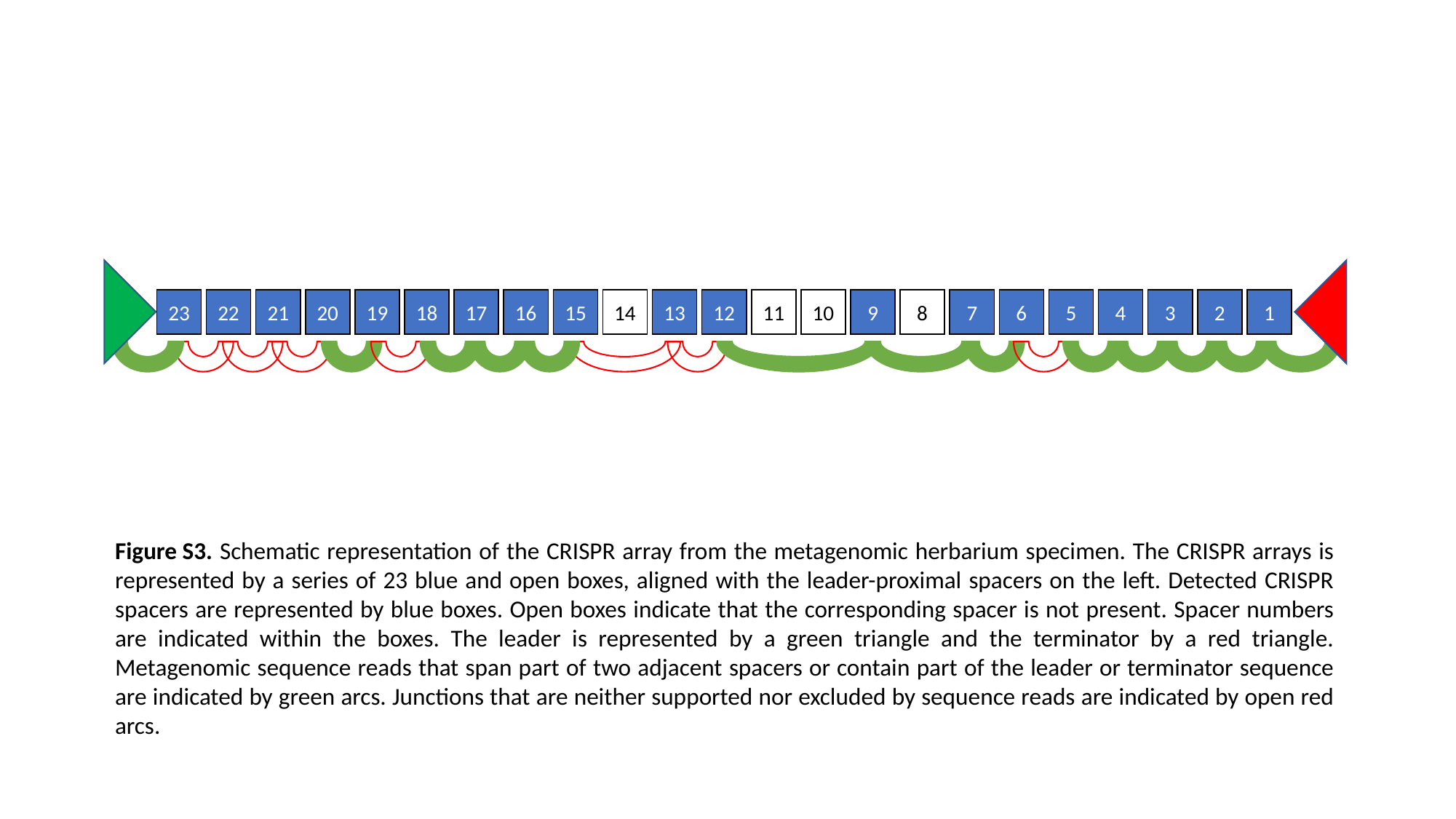

23
22
21
20
19
18
17
16
15
14
13
12
11
10
9
8
7
6
5
4
3
2
1
Figure S3. Schematic representation of the CRISPR array from the metagenomic herbarium specimen. The CRISPR arrays is represented by a series of 23 blue and open boxes, aligned with the leader-proximal spacers on the left. Detected CRISPR spacers are represented by blue boxes. Open boxes indicate that the corresponding spacer is not present. Spacer numbers are indicated within the boxes. The leader is represented by a green triangle and the terminator by a red triangle. Metagenomic sequence reads that span part of two adjacent spacers or contain part of the leader or terminator sequence are indicated by green arcs. Junctions that are neither supported nor excluded by sequence reads are indicated by open red arcs.
